# Real-Time Monitoring on the Chinese Giant Salamander Using RPA-LFD

**DOI:** 10.3390/ijms25094946

**Published:** 2024-05-01

**Authors:** Lanxin Ling, Linyan Liang, Huifang Wang, Xiaolong Lin, Chenhong Li

**Affiliations:** 1 Engineering Research Center of Environmental DNA and Ecological Water Health Assessment, Shanghai Ocean University, Shanghai 201306, China; 2 Shanghai Universities Key Laboratory of Marine Animal Taxonomy and Evolution, Shanghai Ocean University, Shanghai 201306, China; 3 College of Food Science and Technology, Shanghai Ocean University, Shanghai 201306, China

**Keywords:** *Andrias davidianus*, recombinase polymerase amplification, lateral flow strip, real-time monitoring, environmental DNA, conservation

## Abstract

The Chinese giant salamander (*Andrias davidianus*), listed as an endangered species under “secondary protection” in China, faces significant threats due to ecological deterioration and the expansion of human activity. Extensive field investigations are crucial to ascertain the current status in the wild and to implement effective habitat protection measures to safeguard this species and support its population development. Traditional survey methods often fall short due to the elusive nature of the *A. davidianus*, presenting challenges that are time-consuming and generally ineffective. To overcome these obstacles, this study developed a real-time monitoring method that uses environmental DNA (eDNA) coupled with recombinase polymerase amplification and lateral flow strip (RPA-LFD). We designed five sets of species-specific primers and probes based on mitochondrial genome sequence alignments of *A. davidianus* and its close relatives. Our results indicated that four of these primer/probe sets accurately identified *A. davidianus,* distinguishing it from other tested caudata species using both extracted DNA samples and water samples from a tank housing an individual. This method enables the specific detection of *A. davidianus* genomic DNA at concentrations as low as 0.1 ng/mL within 50 min, without requiring extensive laboratory equipment. Applied in a field survey across four sites in Huangshan City, Anhui Province, where *A. davidianus* is known to be distributed, the method successfully detected the species at three of the four sites. The development of these primer/probe sets offers a practical tool for field surveying and monitoring, facilitating efforts in population recovery and resource conservation for *A. davidianus*.

## 1. Introduction

*Andrias davidianus* is listed under “Class II protection” by the National Forestry and Grassland Administration of China (2021) and classified as a critically endangered species by the International Union for Conservation of Nature (IUCN) [1,2]. Belonging to the order Amphibia, suborder Caudata, and family Cryptobranchidae, it is the largest amphibian in the world and endemic to China. Historically, this species was widespread across the mountainous areas of the Yellow River Basin, the Yangtze River Basin, and the Pearl River system [3,4,5], particularly inhabiting water systems that include underground rivers and surface aquatic networks in limestone and karst regions [6]. In recent decades, the wild populations of *A. davidianus* have sharply declined due to continuous environmental deterioration and poaching within its natural habitat, leading to infrequent encounter rates [7,8]. Conducting field investigations to understand the distribution of *A. davidianus* populations is crucial for protecting their habitats and formulating effective conservation strategies. Traditional population monitoring methods of *A. davidianus* primarily involve visual surveys, such as trapping, rock turning, nocturnal snorkeling, and spotlighting surveys [9,10]. However, the rare and elusive nature of *A. davidianus*, which typically resides in inaccessible environments like riverbanks or underground river caves, renders these methods largely ineffective [11,12]. Consequently, the aforementioned approaches often fail to achieve the desired outcomes in practical applications.

Environmental DNA (eDNA) encompasses DNA fragments isolated from environmental samples, which include a diverse array of genetic material from microorganisms, animals, plants, and other species [13]. DNA barcoding is an emerging technology that uses unique DNA sequences to quickly and accurately identify species. This method typically relies on conventional PCR thermal cycling [14,15]. After amplification, species can be identified through gel electrophoresis, or alternatively, classified using second-generation sequencing. This sequencing also provides haplotype information, offering detailed genetic insights into populations and aiding further studies of target species. While agarose gel electrophoresis is widely used for species identification, it demands specific electrophoresis equipment and the entire process, from gel preparation to observation, takes at least one hour. Moreover, the sequencing method involves a more complex procedure, extending from library preparation to data analysis, and invariably requires a laboratory setting [15,16,17,18]. To overcome these limitations, especially in low-resource or field settings, recombinase polymerase amplification (RPA) has been developed [19]. RPA offers a robust, rapid method for DNA amplification that does not require the extensive infrastructure typical of PCR-based techniques, facilitating more flexible and immediate applications in diverse environments.

Recombinase polymerase amplification (RPA) is an isothermal amplification technology that leverages the T4 phage nucleic acid replication mechanism. The RPA reaction primarily utilizes the recombinant enzyme UvsX, loading factor UvsY, single-stranded binding protein SSB, and strand displacement DNA polymerase Bsu, all derived from the T4 phage. The amplification of the template is carried out based on the T4 phage nucleic acid replication mechanism [19,20]. This technique allows for the amplification of the template to be completed efficiently within a brief timeframe, typically 20 to 30 min, and requires maintaining a consistent temperature, thus eliminating the need for thermocycling [21]. Furthermore, when RPA is combined with a lateral flow dipstick (LFD), it allows for rapid result visualization within minutes without complex procedures, making it ideal for field applications [22,23,24]. The utility of RPA technology spans various fields, owing to its fast detection speed, high sensitivity, and simplicity of operation [25,26,27,28]. Research by Wang and Fuller et al. has demonstrated that PCR primers used in RPA yield a higher analytical sensitivity of detection compared to their use in traditional PCR [29,30]. Numerous studies have applied RPA in clinical and on-site sample testing, comparing it to standard methods, primarily PCR. These studies have generally found that while RPA might occasionally produce false negatives, it rarely results in false positives [31,32].

To date, RPA technology has not been applied to the identification of *A. davidianus*. This study pioneers a rapid detection method for *A. davidianus* using the recombinant polymerase amplification combined with a lateral flow dipstick (RPA-LFD), significantly enhancing the investigation and monitoring of its wild populations. The development of this novel method not only improves the accuracy and efficiency of *A. davidianus* identification, but also introduces innovative approaches for scientific research and practical applications in conservation and related fields.

## 2. Results

### 2.1. Selecting Optimal Primer/Probe Sets for RPA Reaction

**Figure 1:**
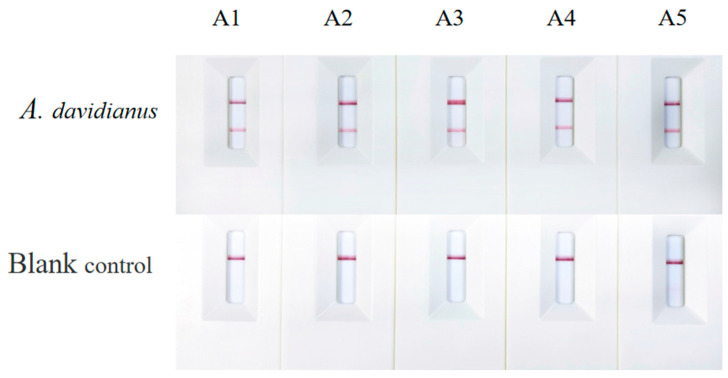
Results of the recombinant polymerase amplification combined with a lateral flow dipstick (RPA-LFD) detection using the genomic DNA of *Andrias davidianus* and a blank control with primer/probe sets. A1 to A5 correspond to W1, W2, W3, W4, and W5, respectively.

All five pairs of the primer/probe sets successfully amplified the genomic DNA of *A. davidianus*. However, the blank control of primer/probe W5 exhibited weak positive results (Figure 1), indicating potential cross-reactivity between the primers and probe of W5, leading to a false-positive result. Based on these findings, primer/probe sets W1, W2, W3, and W4 were selected for use in subsequent experiments.

### 2.2. Specificity and Sensitivity Test on the Primer and Probe Sets

**Figure 2:**
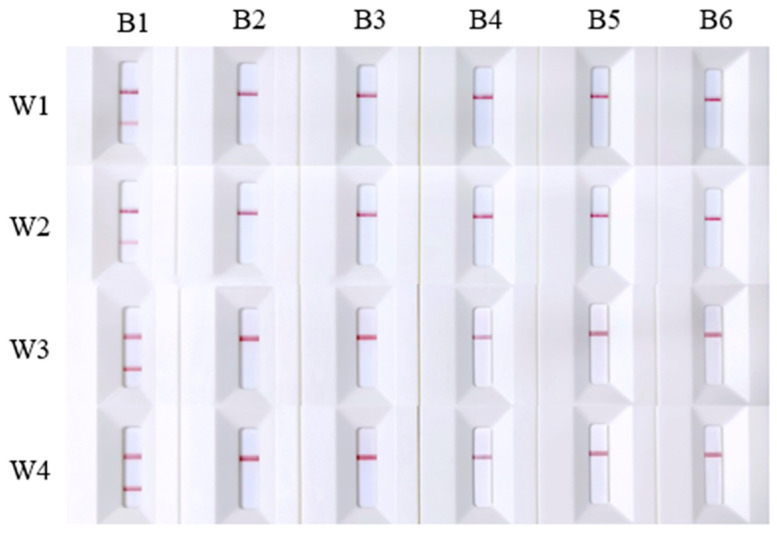
Results of the specificity tests with primer/probe sets: W1, W2, W3, and W4. Labels B1 to B6 represent *Andrias davidianus*, *Paramesotriton zhijinensis*, *Pseudohynobius flavomaculatus*, *Blue-tail Cynops*, *Cynops orientalis*, and water, respectively.

The results of the RPA-LFD test showed that *A. davidianus* DNA tested positive with primer/probe sets W1–W4, while other caudata species and the blank control yielded negative results (Figure 2). The experimental findings suggest that the primer/probe sets W1-W4 exhibit good specificity and are suitable for the field monitoring of *A. davidianus*.

**Figure 3:**
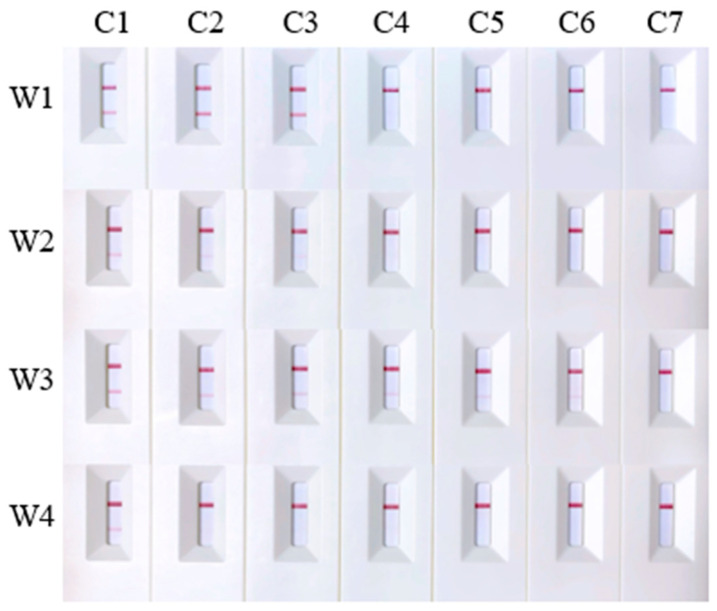
Sensitivity test results for primer/probe sets W1–W4. C1 to C7 represent DNA samples of *Andrias davidianus* at concentrations of 1000 ng/mL, 100 ng/mL, 10 ng/mL, 2 ng/mL, 1 ng/mL, and 0.1 ng/mL, respectively, along with the negative control.

**Figure 4:**
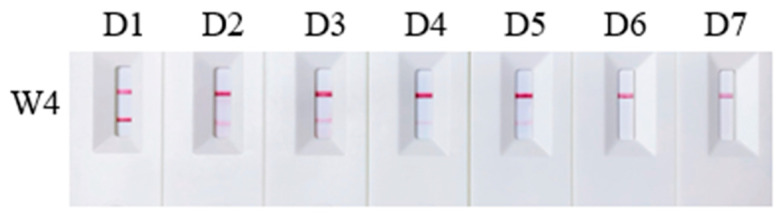
Sensitivity tests for primer/probe W4 using a 10-fold dilution ratio. D1 to D7 represent DNA samples of *Andrias davidianus* at concentrations of 1000 ng/mL, 100 ng/mL, 10 ng/mL, 2 ng/mL, 1 ng/mL, and 0.1 ng/mL, respectively, as well as the negative control.

Four primer/probe sets, W1–W4, were selected based on their specificity to evaluate their sensitivity. The genomic DNA of *A. davidianus* was serially diluted to concentrations of 1000 ng/mL, 100 ng/mL, 10 ng/mL, 2 ng/mL, 1 ng/mL, and 0.1 ng/mL for testing. The results revealed that primer/probe sets W1 and W2 failed to yield positive results at concentrations below 10 ng/mL, establishing 10 ng/mL as their minimum detection limit. In contrast, primer/probe W3 maintained good sensitivity, detecting positive results at concentrations as low as 0.1 ng/mL. However, primer/probe W4 only detected the target at a concentration of 1000 ng/mL, indicating lower sensitivity (Figure 3). To enhance sensitivity, we modified the dilution protocol by extracting 10 μL of reaction solution from the original 50 μL reaction system and added 90 μL sterile deionized water instead of 190 μL for a 10-fold dilution. The adjustment enabled primer/probe W4 to detect template DNA at a concentration of 2 ng/mL (Figure 4). These findings suggest that the dilution ratio of the final reaction mixture significantly impacts detection sensitivity. Lowering the dilution ratio can effectively increase the sensitivity of the detection results when the concentration of template DNA is minimal.

### 2.3. Testing the Primer/Probe Sets on Mock Environmental Samples

**Figure 5:**
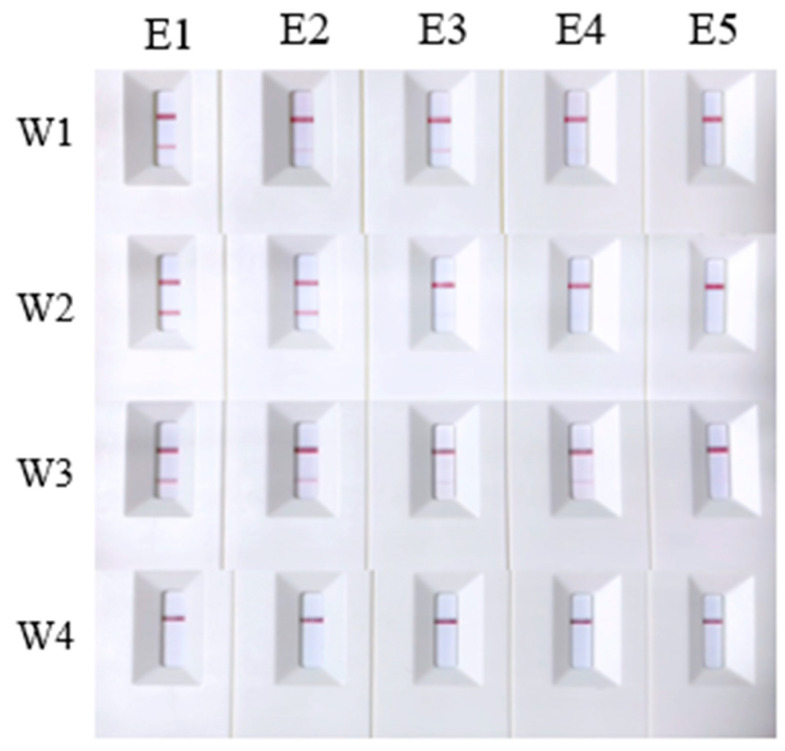
Test results of primer/probe sets W1–W4 on DNA extracted with different methods. E1 to E5 represent the detection results of genomic DNA, eDNA extracted by the regular CTAB method, eDNA extracted using Chelex 100, eDNA extracted using the rapidly CTAB, and the negative control, respectively.

**Figure 6:**
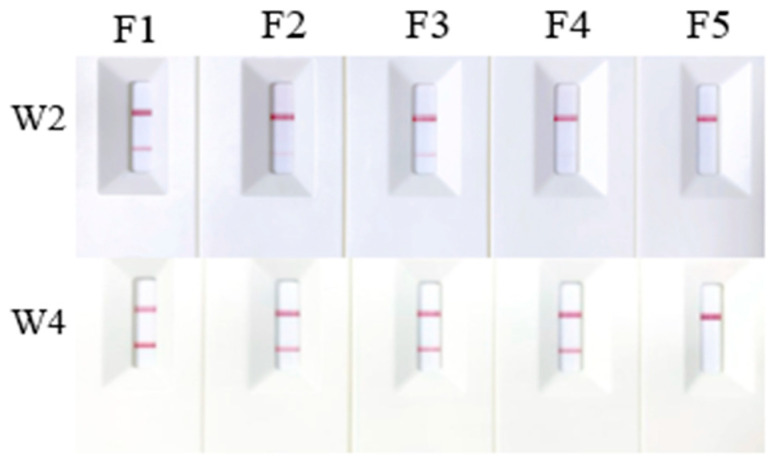
Testing of primer/probes W2 and W4 on DNA diluted at a 10-fold ratio. F1 to F5 represent the detection results of genomic DNA, eDNA extracted by the regular CTAB method, eDNA extracted using Chelex 100, eDNA extracted using the rapidly CTAB, and the negative control, respectively.

The results showed that W1 and W3 successfully detected eDNA obtained using all three extraction methods: the original CTAB method, Chelex 100, and the modified rapid CTAB method. Although primer/probe set W2 also yielded positive results across all methods, the bands were faint for samples extracted using Chelex 100 and the rapid CTAB method. In contrast, W4 was only able to detect eDNA extracted using the regular CTAB method and failed with the rapid method (Figure 5). Given the relatively low concentration of eDNA in the water samples and varying sensitivities of primers, we diluted the reaction products of W2 and W4 by a factor of ten and then tested them with the strips. This adjustment led to noticeable positive results for both primer/probe sets across the three extraction methods (Figure 6), reinforcing the conclusions discussed in Section 2.2.

### 2.4. Field Tests

**Figure 7:**
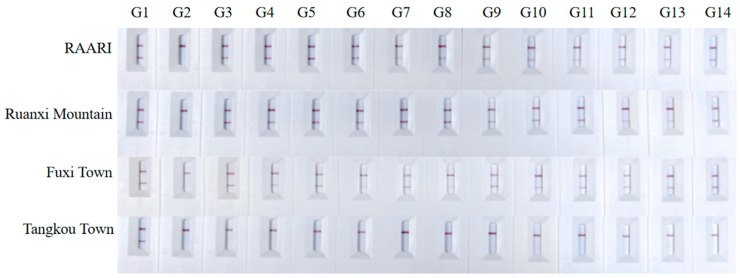
Field testing of the recombinant polymerase amplification combined with a lateral flow dipstick (RPA-LFD) with primer/probe sets on *Andrias davidianus*. Testing was conducted on water samples collected from the Rare Aquatic Animal Research Institute (RAARI), Ruanxi Mountain, Tangkou Town. G1 is designated as the positive control, G2 as the negative control, and G3 to G14 represent three replicates of detections using primer/probe sets W1–W4.

Referring to the results of the methods described in Section 4.5 and Section 2.3, both rapid-CTAB and Chelex 100 have proven effectiveness and efficiency in extracting DNA from water samples. However, the Chelex 100 method stands out for its rapidity and convenience, making it especially suitable for field applications. Consequently, we selected the Chelex 100 method to extract the eDNA samples collected in the field for testing. Given that eDNA concentrations in field might be lower than those in laboratory settings, we adjusted the RPA system accordingly. Specifically, we replaced all sterilized deionized water in the RPA system with a DNA sample, using 13.5 μL of template DNA instead of the standard 2 μL in the 50 μL RPA reaction mix. Additionally, we diluted the RPA product by a factor of ten before applying it dropwise to the test strip for observation. The results showed that *A. davidianus* was detected by all four primer/probe sets at three sampling sites: RAARI, Ruanxi Mountain, and Fuxi Town. However, no positive results were obtained from samples collected from Tangkou Town (Figure 7).

## 3. Discussion

*Andrias davidianus*, the largest extant amphibian globally, is a unique and rare species native to China. This creature not only holds substantial scientific and ecological value but also possesses considerable economic importance, being utilized in both food and traditional Chinese medicine practices [33]. Unfortunately, its populations have been declining over recent decades due to ecological degradation and overfishing. This decline has led to a reduced geographical distribution and poses an imminent threat of extinction. Despite protective measures enacted by the Chinese government, the conservation status of its wild population remains precarious, with the International Union for Conservation of Nature (IUCN) listing it as critically endangered in its 2021 assessment [34]. Artificial breeding and reintroduction initiatives are pivotal for the protection of *A. davidianus*. However, augmenting wild populations with artificially bred individuals might increase the population size but could potentially impact the genetic diversity of the species. Yan et al. [35] identified five haplotypes and suggested the existence of at least five subspecies within *A. davidianus*. Chai et al. [36] discovered a new species, *Andrias jiangxiensis* sp. nov., which supports Yan’s findings, indicating at least six subspecies within *A. davidianus*. Poor taxonomy can drive species to extinction, as some cryptic species may vanish due to unrecognized conservation needs [35]. Hence, the indiscriminate release of farmed *A. davidianus* into the wild without genetic considerations could dilute the evolutionary distinctiveness of native populations and increase the risk of extinction through genetic homogenization. Investigating cryptic species within the *A. davidianus* population can help protect native species without genetic contamination, preserve the evolutionary uniqueness of the native population, enhance the population’s genetic diversity, and decrease the risk of extinction. Effective on-site monitoring is crucial for the successful implementation of conservation strategies. Although the method we developed in this study was unable to identify cryptic species within the *A. davidianus* populations, it can swiftly confirm the presence of *A. davidianus* within the investigation area, laying the groundwork for subsequent sequencing and identification of cryptic species.

Traditional monitoring methods, while thorough, involve high costs and cumbersome procedures that hinder real-time assessment of the species’ distribution and population dynamics. A study by Liu et al. [2] observed populations of *A. davidianus* released into Gutianshan National Nature Reserve in Zhejiang province using both traditional methods and eDNA techniques. While the integration of eDNA with PCR technologies enabled non-invasive monitoring, this approach proved as time-consuming as traditional methods. The necessity for a thermocycler for PCR amplification and subsequent identification by gel electrophoresis precludes real-time monitoring of the *A. davidianus* population in the field. Given these challenges, there is a pressing need to explore more practical methods that enable convenient, sensitive, and real-time monitoring of *A. davidianus* populations in their natural habitats.

Compared to other isothermal amplification methods like loop-mediated isothermal amplification of DNA (LAMP), RPA offers several advantages. The design of an RPA primer is straightforward, and its reaction kinetics are rapid. Unlike LAMP, RPA does not require a thermocycler, making it more adaptable for field use. Additionally, the inclusion of probes in the RPA reaction enhances detection precision and allows for the direct use of the amplification product in subsequent analyses [37,38]. RPA products can be detected in various ways, among which the LFD is particularly valued for its rapid field detection capabilities. The integration of RPA technology with LFD, which delivers visual results quickly, is highly important for achieving rapid and portable field detection [39]. Furthermore, the capability to extract DNA without complex instrumentation is a key advantage of using RPA-LFD for swift, on-site detection, and can be efficiently executed under consistent temperature conditions. For instance, Zou et al. [40] established a reverse transcription recombinase polymerase amplification combined with a lateral flow strip (SMYEV-RT-RPA-LF) to diagnose strawberry mild yellow edge virus (SMYEV) in strawberries. They employed the NF-2 kit (Shenzhen Braveds Biotech Co., Ltd., Shenzhen, China) to extract RNA from strawberry leaves, using a lysis supernatant—after a five-fold serial dilution—as a direct template for RT-PCR or SMYEV-RT-RPA-LF. Similarly, Mayran et al. [41] crafted an approach that combines isothermal RPA with direct visual detection on a lateral flow assay (LFA) to detect Hepatitis B virus (HBV). They effectively extracted DNA from HBV-positive plasma samples using Chelex 100, facilitating subsequent experiments. The aforementioned studies, along with our own, have utilized straightforward DNA extraction methods and the RPA-LFD approach, demonstrating its efficacy for rapid diagnosis in field settings. The simplicity and speed of these methods make them invaluable tools for on-site applications, confirming the practical utility of RPA technology in diverse diagnostic scenarios.

The efficacy of RPA technology hinges significantly on the design of its primers and probes. Currently, there is no comprehensive software dedicated to the entire process of designing RPA primers and probes. Consequently, the design of these components requires meticulous manual examination and strict adherence to established principles to prevent false-positive results due to unintended hybridization of primers and probes [42]. For example, the primer/probe set W5 designed in our experiment was found to yield weak positive results in blank controls and was therefore excluded from further use. It is also important to note that adverse primer interactions, such as hairpin structure or primer dimer formations, may occur within the RPA reaction environment. These interactions can serve as substrates for DNA polymerase, leading to the synthesis of lower molecular weight DNA, known as primer noise. Designing primers and probes with 5–9 mismatched base pairs can significantly reduce the formation of primer dimers, thus decreasing primer noise without compromising the efficiency of RPA analysis [43,44]. Thus, to mitigate such issues, it is also advisable for researchers to design multiple sets of primers and probes initially to allow for thorough screening.

The sensitivity test results clearly demonstrate that the dilution ratio of reaction products substantially affects detection outcomes. Consequently, to enhance sensitivity, especially when the concentration of eDNA in the field is low, we recommend adjusting the dilution ratio. In our RPA system, the total volume of DNA template and sterile deionized water typically equals 13.5 μL. An effective strategy for boosting sensitivity in cases of low eDNA concentration involves using a 13.5 μL sample without any added water. For field testing using RPA-LFD, employing a portable thermostatic device, such as temperature-controlled thermos, is recommended to maintain consistent reaction temperatures. Research indicates that optimizing key reaction system parameters—such as temperature, time, and magnesium acetate concentration—can significantly enhance the efficiency of RPA amplification. This optimization is crucial for addressing issues like false positives in RPA-LFD results [45]. Thus, we recommend that the primers and probes employed in this study be further optimized concerning their reaction temperatures and times to enhance their effectiveness in practical applications. Additionally, it is essential for researchers to carefully select a pristine and unoccupied environment and to rigorously maintain sterile conditions to minimize the risk of contamination. By continuously refining these reaction parameters, the accuracy and reliability of the outcomes can be maximized, ensuring the highest level of precision in experimental results.

The field survey was carried out on Huangshan Mountain, where water samples were collected from four distinct locations. Traces of *A. davidianus* were detected in three of these locations: RAARI, Ruanxi Mountain, and Fuxi Town, while Tangkou Town showed no evidence of the species. The presence of *A. davidianus* near the breeding farm (RAARI) was anticipated due to the likely high concentration of eDNA from the species in the nearby water. The detections at Ruanxi Mountain and Fuxi Town are consistent with previous reports of *A. davidianus* in these areas. Conversely, the absence of *A. davidianus* in Tangkou Town may be due to the dense population and severe water pollution, which render the habitat unsuitable for *A. davidianus*.

In conclusion, our experiment demonstrated the feasibility of combining RPA-LFD with a rapid DNA extraction method. The RPA-LFD method established in this study provides a quick and effective means of monitoring *A. davidianus* in the wild. This approach is likely to have a positive impact on the population recovery and resource conservation of this endangered species.

## 4. Materials and Methods

### 4.1. DNA Samples

Tissue samples of *A. davidianus* were collected from farm-bred individuals at Xuejiazhuang Aquaculture Farm (Hanzhong, Shangxi Province, China). Control samples from other caudata species, such as *Paramesotriton zhijinensis*, *Pseudohynobius flavomaculatus*, Blue-tail *Cynops*, and *Cynops orientalis* were also collected. All procedures involving animals were conducted in accordance with the “Ethical Standards of the Shanghai Ocean University (2020)”. Genomic DNA of *A. davidianus* and the other caudata species was extracted using an Ezup column animal genomic DNA extraction kit (Sangon Bioengineering Co., Ltd., Shanghai, China). The concentration of DNA in these samples was measured using a NanoDrop 3300 Fluorospectrometer (Thermo Fisher Scientific, Wilmington, DE, USA) before being stored at −20 °C for subsequent use.

### 4.2. Design of Primers and Probes

Mitochondrial genome sequences of *A. davidianus* and its close relatives were retrieved from NCBI (Table 1). PrimedRPA [46] was used for an initial comparison between *A. davidianus* and other species to identify conserved regions within the gene sequence of *A. davidianus*. Subsequently, MEGA-X [47] and visual inspection were employed to design specific primers and probes for *A. davidianus*. The primer design adhered to the following criteria: primer length of 30–35 base pairs; GC content between 30–70%; amplified fragment length of 150–300 base pairs; avoidance of secondary structure formation in the amplified fragments; and the 5′ end of the downstream primer was biotin-labeled. A 46–52 base pair RPA probe was then designed between the upstream and downstream primers. Modifications included a FAM group at the 5′ end, a dSpacer (oxolane, THF) at the recognition site, and a C3 spacer at the 3′ end. [21,40,42]. The primers were synthesized by GENEWIZ Biotechnology Co., Ltd. (Suzhou, China) and the probe by Sangong bioengineering. In total, five sets of primers and probes were designed (Table 2).

### 4.3. Test of Specificity

Genomic DNA from *A. davidianus* and other caudata species (*P. zhijinensis*, *P. flavomaculatus*, *B. cynops*, and *C. orientalis*) served as templates, while sterile deionized water was used as the negative control. RPA amplification was performed according to the instructions provided by the DNA isothermal rapid amplification kit (AMP Future Biotechnology Co., Ltd., Shanghai, China). The reaction temperature was set at 37 °C with a duration of 10 min. The RPA reaction mixture included 29.4 μL of Buffer A, 2 μL of 10 μM upstream and downstream primers, 0.6 μL of 10 μM probe, 2–13.5 μL DNA template (topped up to 13.5 µL with sterile deionized water), and 2.5 μL of Buffer B. Afterwards, the components were rapidly vortexed and centrifuged. The mixture was then incubated in a 37 °C metal bath for 10 min. Following incubation,10 μL of the reaction solution was diluted with 190 μL of sterile deionized water, and 50 μL of this diluted solution was applied to the HybriDetect strip (AMP Future Biotechnology Co., Ltd., Shanghai, China) after vigorous shaking and thorough mixing. The results were visually inspected.

### 4.4. Test of Sensitivity

The genomic DNA of *A. davidianus* was serially diluted to concentrations of 1000 ng/mL, 100 ng/mL, 10 ng/mL, 2 ng/mL, 1 ng/mL, and 0.1 ng/mL using sterile deionized water. These diluted DNA samples were tested using the same procedure as described in the specificity test, with sterile deionized water acting as the negative control. The sensitivity of the primers and probes was determined based on the lowest concentration of diluted DNA that can be detected by the RPA-LFD method.

### 4.5. Examination on Mock Environmental Samples

To assess the effectiveness of the primer/probe sets on environmental samples, water was collected from a 40 L aquarium housing a 0.5 kg farm-bred *A. davidianus*. Three water samples were processed as replicates, each being filtered through a 47 mm diameter polycarbonate filter membrane with a 2 μm pore size. Distilled water, filtered in the same manner, served as a negative control.

Given the constraints of field conditions, it is essential to develop a rapid DNA extraction method for water samples. Alongside the traditional CTAB extraction, we evaluated two accelerated eDNA extraction methods: modified CTAB and Chelex 100, aiming to reduce the extraction time to under 40 min.

The optimization of the CTAB extraction method proceeded as follows: the water sample was first pumped through the filter membrane, which was then transferred to a centrifuge tube containing 700 μL of CTAB and 700 μL of phenol chloroform, and vortexed for 2 min. The samples were then spun in a portable centrifuge at the highest speed for 10 min. The supernatant was carefully collected and mixed with 500 μL of iced isopropyl alcohol and 250 μL of 5 M sodium chloride, then centrifuged again for 10 min. After removing the supernatant, the remaining pellet was washed twice with 150 μL of 70% ethanol, followed by a 5 min centrifugation each time. The ethanol was discarded, the tube opened, and the pellet was air-dried for 5 min. Finally, 100 μL of TE solution was added to re-suspend the pellet.

The Chelex 100 extraction method proceeded was follows: the filter membrane was cut into pieces and placed in a centrifuge tube, to which 500 μL 10% (*w*/*v*) Chelex 100 and 20 μL of protease K were added. The mixture was then incubated in a metal bath at 56 °C for 10 min, with the temperature subsequently raised to 99 °C and maintained for 15 min. After centrifugation for 10 min, the supernatant was retained for further analysis.

The extracted DNA samples were amplified using RPA and checked with an LFD strip to determine presence or absence of *A. davidianus* in the water samples, following the method described in Section 4.3.

### 4.6. Application of the Developed Primes/Probes and RPA-LFD Method in Field

**Figure 8:**
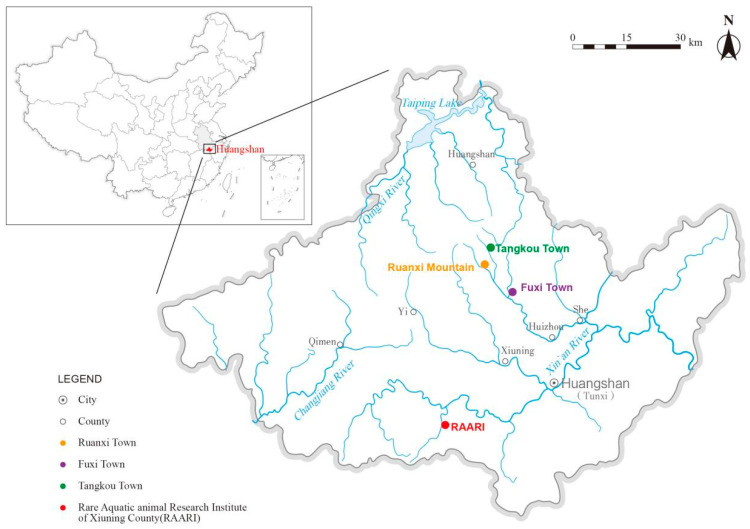
Sampling map for on-site testing of the recombinant polymerase amplification combined with a lateral flow dipstick (RPA-LFD) method in detecting *Andrias davidianus*.

To assess the feasibility of the methods under field conditions, surveys were conducted at four sites in Huangshan City, Anhui Province, China, from 28 May to 1 June 2023 (Figure 8; Table S1). These sites are located along various headwater branches of the Xin’an River drainage system. Notably, the Rare Aquatic Animal Research Institute of Xiuning County, Huangshan City (RAARI), is situated near a farm breeding *A. davidianus*. Sampling was strategically performed at the farm’s outlet to ensure the detection of *A. davidianus*, thus serving as a positive control. At each site, including 100 m upstream and downstream of the sampling location, 2 L of water samples were collected. These samples were pooled, and 1 L of the combined sample was filtered for each of the three sample replicates. The DNA extraction method followed the Chelex 100 method outlined in Section 4.5. Subsequently, the extracted DNA samples were subjected to RPA amplification and were analyzed using LFD strips. All field equipment used was portable, including a portable vortex, portable centrifuge, and metal baths, which facilitated easy transportation and usage in various field settings.

## Figures and Tables

**Table 1:** NCBI numbers of *Andrias davidianus* and its close relatives.

Species	NCBI Accession Numbers
*Andrias davidianus*	NC_004926.1
*Andrias japonicus*	NC_007446.1
*Randon sibiricus*	NC_004021.1
*Hynobius unisacculus*	NC_045210.1
*Hynobius maoershanensis*	NC_023789.1
*Hynobius yangi*	NC_013825.1
*Hynobius guabangshanensis*	NC_013762.1
*Hynobius quelpaertensis*	NC_010224.1
*Hynobius amjiensis*	NC_008076.1
*Hynobius chinensis*	NC_008088.1
*Hynobius arisanensis*	NC_009335.1
*Hynobius leechii*	NC_008079.1
*Hynobius formosanus*	NC_008084.1
*Pseudohynobius puxiongensis*	NC_020634.1
*Onychodactylus zhaoermii*	NC_026854.1
*Onychodactylus zhangyapingi*	NC_026853.1
*Onychodactylus fischeri*	NC_008089.1
*Batrachuperus yenyuanensis*	NC_012430.1
*Batrachuperus gorganensis*	NC_008091.1
*Batrachuperus mustersi*	NC_008090.1
*Batrachuperus tibetanus*	NC_008085.1
*Batrachuperus pinchonii*	NC_008083.1
*Batrachuperus londongenensis*	NC_008077.1
*Salamandrella keyserlingii*	NC_008082.1
*Liua shihi*	NC_008078.1
*Liua tsinpaensis*	NC_008081.1
*Pachyhynobius shangchengensis*	NC_008080.1

**Table 2:** Recombinant polymerase amplification combined with a lateral flow dipstick (RPA-LFD) method amplifying primers and probes for *Andrias davidianus*.

Name	Sequences (5′-3′)
W1-F	CTAACCACATCCCATAATATATCAAACTCTAA
W1-R	Biotin-CTCTTGGTCTCTTATCCTAAGTCTTTATATTA
W1	FAM-AACCTTTATATTAATATCATTATTAATCATCCTC/dSpacer/TCTCTCATATTACTCCCT-C3spacer
W2-F	TCTAAGTGTAAGTATAAATCAAAACGAACCC
W2-R	Biotin-GAGTTCCTTCTTTGACTTTTAATCTTTCTT
W2	FAM-GAACCATATTGAAGGTAACATCTATTTTAAGCAAG/dSpacer/AAATTTTGATTC -C3spacer
W3-F	CACCCATTACACGTCTTATCATTATCAGCCC
W3-R	Biotin-CCTAGCATGAAGGTTGTGTAAAATGTAAAATA
W3	FAM-AGGTCTTGAGTGAGCCCAATAAGTATTTAGTCC/dSpacer/AATAAAGACCGCTGA-C3spacer
W4-F	CAGTAATAACATTAAAAGTCGGTAAATCCC
W4-R	Biotin-CCTAGCATGAAGGTTGTGTAAAATGTAAAATA
W4	FAM-TCAATGACGAAAGTAATTCTAGATAATGACC/dSpacer/CCACGAAAATTAGGTT-C3spacer
W5-F	TTCAAATCCTCTTCTTAG
W5-R	Biotin-GGACGGATTGGTTCTTTAATAAATAGTTTT
W5	FAM-TTACGAAAAGGGCCAAACATTGTAGGCCCTGCC/dSpacer/GGCATTCTTCAACCAT-C3spacer

## Data Availability

Data is contained within the article and Supplementary Materials.

## Supplementary Materials

The supporting information can be downloaded at: https://www.mdpi.com/article/10.3390/ijms25094946/s1.
