# Supplementary material for: Real-Time Monitoring on the Chinese Giant Salamander Using RPA-LFD
Source: Int J Mol Sci. 2024 May 1;25(9):4946. doi: 10.3390/ijms25094946 (PMC11084824; doi:10.3390/ijms25094946)
Supplement: Supplementary file 1 [file ijms-25-04946-s001.zip › supplementary.pdf]

Table S1. Sampling sites for testing RPA-LFD method in the field

Sites	Longitudes	Latitudes
Rare Aquatic Animal Research Institute (RAARI)	117°59′29"E	29°38′32"N
Ruanxi Mountain	118°7′46"E	30°17′31"N
Fuxi Town	118°12′55"E	29°57′41"N
Tangkou Town	118°10′1"E	30°39′36"N
